# Supplementary material for: CXCR5 guides migration and tumor eradication of anti-EGFR chimeric antigen receptor T cells
Source: Mol Ther Oncolytics. 2021 Jul 16;22:507–17. doi: 10.1016/j.omto.2021.07.003 (PMC8433065; doi:10.1016/j.omto.2021.07.003)
Supplement: Document S1. Figures S1 and S2 [file mmc1.pdf]

OMTO, Volume *22*

Supplemental information

CXCR5 guides migration and tumor eradication of anti-EGFR chimeric antigen receptor T cells

Guangchao Li, Jintao Guo, Yanfang Zheng, Wen Ding, Zheping Han, Lingyu Qin, Wenjun Mo, and Min Luo

Supplementary Figure 1

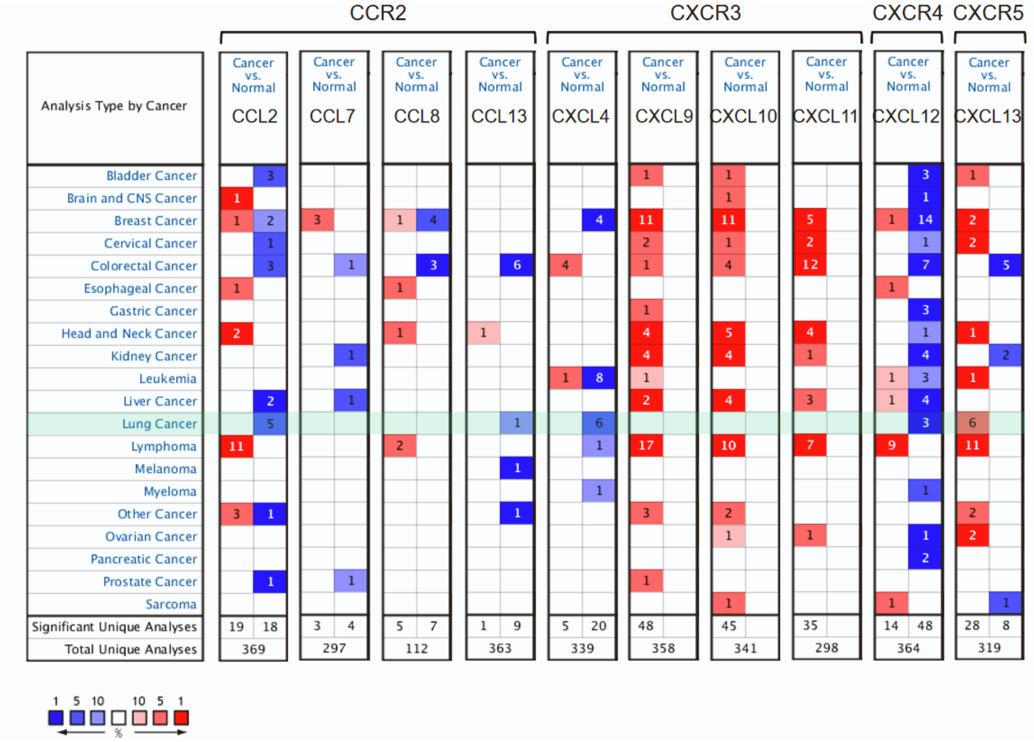

Figure S1. The expressions of multiple chemokines in the normal and lung cancer.

The mRNA expression levels of chemokines in various cancers were analyzed using the online Oncomine database (Threshold for FC>2, p<0.05, and rank=top10).

Supplementary Figure 2

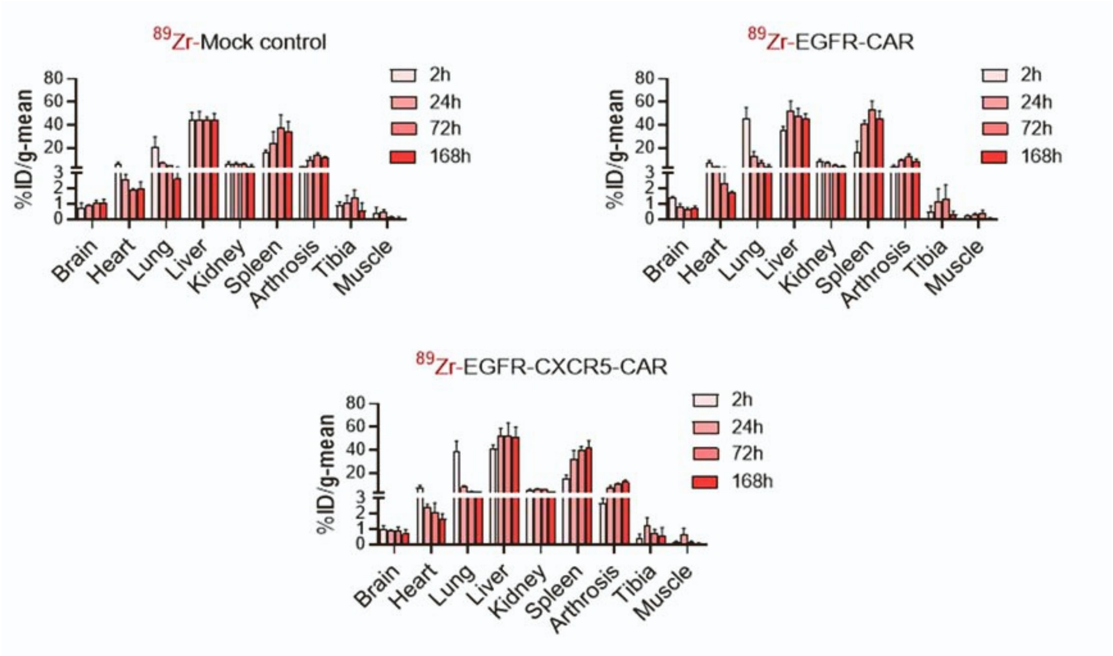

Figure S2. Micro PET/CT analysis of the 89Zr-Oxine labeled CAR-T cells tracking in vivo.

After a single intravenous injection of ^{89}Zr-CAR-T cells in tumor-bearing mice, a small animal PET/CT scan was performed to delineate the regions of interest and obtain the radiation uptake values to study the tissue distribution characteristics of CAR-T cells. Radio-isotopes were imaged on tumor bearing mice over 7 days (n=3).
