# Supplementary material for: CXCR5 guides migration and tumor eradication of anti-EGFR chimeric antigen receptor T cells
Source: Mol Ther Oncolytics. 2021 Jul 16;22:507–17. doi: 10.1016/j.omto.2021.07.003 (PMC8433065; doi:10.1016/j.omto.2021.07.003)
Supplement: Document S2. Article plus supplemental information [file mmc2.pdf]

Molecular Therapy
Oncolytics
Original Article

CXCR5 guides migration and tumor eradication of anti-EGFR chimeric antigen receptor T cells

Guangchao Li,[1,4] Jintao Guo,[1,4] Yanfang Zheng,[2,4] Wen Ding,[1] Zheping Han,[3] Lingyu Qin,[2] Wenjun Mo,[1] and Min Luo[1]

[1]Guangzhou Bio-gene Technology Co., Ltd., Guangzhou, Guangdong Province, China; [2]Department of Oncology, Zhujiang Hospital of Southern Medical University, Guangzhou, Guangdong Province, China; [3]Department of Laboratory Medicine, Central Hospital of Panyu District, Guangzhou, Guangdong Province, China

The efficacy of chimeric antigen receptor (CAR) T is still not optimal for solid tumors, partly due to the lack of T cell infiltration to the tumor site. One promising strategy is to guide T cells through tumor-specific chemokines, provided that the matching chemokine receptors are expressed on T cells. Previous reports showed that, for non-small cell lung cancer (NSCLC) patients, the tumor sites express high levels of chemokine CXCL13, whereas CXCR5, the only receptor for CXCL13, is mainly expressed on B cells and follicle helper T cells. Therefore, we engineered an epidermal growth factor receptor (EGFR) CAR-T cell to express a second receptor CXCR5, to facilitate migration of CAR-T cells to the CXCL13-expressing NSCLC tumors, and to minimize EGFR-CAR-T possible off-tumor, on-target toxicity. We first confirmed CXCL13 expression in NSCLC patient blood and cancer tissues and the absence of CXCR5 expression in normal CD3 T cells. Next, we demonstrated that EGFR-CXCR5-CAR-T cells have similar killing activity as EGFR-CAR-T with a cytotoxicity assay *in vitro*. Furthermore, the *in vitro* Transwell assay and *in vivo* xenograft tumor mouse model were used to confirm that EGFR-CXCR5-CAR-T exhibits a significant increase in T cell infiltration to CXCL13-expressing tumors and eradicates the CXCL13-expressing tumors more efficiently.

INTRODUCTION

The chimeric antigen receptor (CAR) T cell therapy has shown supreme clinical antitumor efficacies in hematological malignancies that express CD19.[1–3] However, the efficacy of CAR-T is still not optimal for solid tumors. Numerous preclinical studies have observed superior efficacies of CAR-T cells against solid tumor models including glioblastoma,[4,5] sarcoma,[6] neuroblastoma,[7] breast cancer,[8] advanced gastric or pancreatic cancer,[9] lung cancer,[10] hepatocellular carcinoma,[11] ovarian cancer,[12] and prostate cancer.[13] However, the clinical outcome is rather poor, and the responses are transient,[14] due to a variety of factors, such as antigen escape, limited tumor infiltration, and restricted CAR-T survival and amplification.

Mounting evidence suggests the classical CAR-T design is not efficient to cure solid tumor. As a result, major efforts have been put in to increase CAR-T cell migration, proliferation, and persistence *in vivo*.[15–17] It is shown that only a small fraction of the transferred T cells can eventually reach the tumor sites despite that a large number of CAR-T cells are infused.[18] One reason for insufficient recruitment of CAR-T cells to tumor is the mismatching of chemokine-chemokine receptor pairs, especially the lack of CXCL9 and CXCL10, the two ligands of chemokine receptor CXCR3 typically expressed on activated T cells.[19,20] Given the criticality of chemokines in lymphocyte migration and homing, engineering the chemokine receptor that pairs with the chemokine produced by tumors may confer CAR-T the tumor-specific homing capability and thus improve clinical safety and efficacy.

The corresponding receptor to CXCL13 is CXCR5 that is mainly expressed on B cells and follicular helper T (T_{fh}) cells,[21,22] which are part of the CD4 T cell population and do not have a direct cytotoxic effect. CXCR5-CXCL13 pair signaling plays an important chemotactic role in bringing the $CXCR5^+$ T_{fh} and B cells to the germinal centers during B cell affinity maturation of antibodies (Abs).[23] Previous studies have shown that 70% of non-small cell lung cancer (NSCLC) patients from highly polluted areas in China exhibited high levels of CXCL13 expression in the tumor tissue.[24] Elevated levels of CXCL13 were also confirmed in the blood of NSCLC patients compared with healthy controls[25].

One study reported that 11 relapsed/refractory NSCLC patients with >50% epidermal growth factor receptor (EGFR) positive in lung tissue immunohistochemistry (IHC) at stage IIB or higher were treated with CAR-T cells targeting at EGFR in combination with chemotherapy pre-conditions.[26] EGFR-CAR-T cell dosages at 0.04 to 2.54 $\times$ 10^7 cells/kg body weight were tolerated by patients with no severe adverse events reported. The clinical outcome was however unsatisfactory: 2 patients achieved partial response, and 5 patients had stable disease.[26]

Based on these findings, we hypothesized that T cells expressing an EGFR-targeting CAR and the CXCR5 receptor will pair with CXCL13 produced by NSCLC tumors, further enhancing the

Received 4 March 2021; accepted 9 July 2021;
https://doi.org/10.1016/j.omto.2021.07.003.

[4]These authors contributed equally

Correspondence: Min Luo, PhD, Guangzhou Bio-gene Technology Co., Ltd., 206 Kaiyuan Blvd., Guangzhou City, Guangdong Province 510530, China.
E-mail: mluo@gzbiogene.com

This is an open access article under the CC BY license (http://creativecommons.org/licenses/by/4.0/).

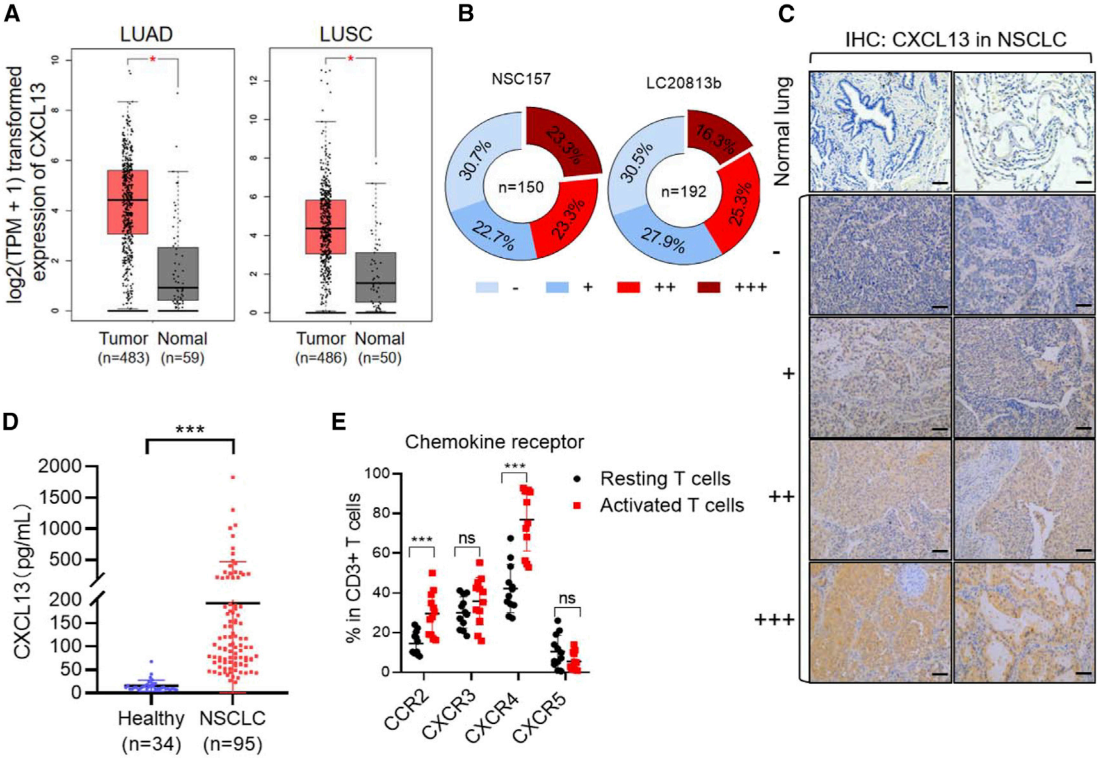

Figure 1. Restricted normal T cell expression of CXCR5 and upregulation of CXCL13 in non-small cell lung cancer (NSCLC)

(A) The expression of CXCL13 in patients with lung adenocarcinoma (LUAD) and lung squamous cell carcinoma (LUSC) using the online tool of GEPIA. (B) CXCL13 protein expressions in NSCLC tissues were confirmed by immunohistochemistry on two tissue microarray slides (NSC157 and LC20813b). The intensity of immunostaining was graded as follows: −, negative; +, weak; ++, moderate; or +++, strong. (C) Expression of CXCL13 by immunohistochemistry. The subpanels show negative expression of CXCL13 (−), weak (+), moderate (++), and strong (+++) expressions of CXCL13 in tumor tissues (×400). (D) ELISA quantification of the level of CXCL13 protein in plasma samples (healthy donors n = 34, NSCLC patient donors n = 95). Single dot represents individual plasma sample. Error bars represent mean ± SD. ***$p < 0.001$. (E) FACS analysis of the expression of different chemokine receptors from resting and activated T cells. Single dot represents individual sample. Error bars represent mean ± SD for each T cell population (n = 12).

T cell migration and cancer cell killing. In this study, we engineered a vector for co-expression of anti-EGFR-CAR and CXCR5. Our experiments, both *in vitro* and *in vivo*, demonstrated that this novel design increases CAR-T cell migration to CXCL13-positive tumor tissues and enhances the ensuing killing of target cells.

RESULTS

Elevated levels of CXCL13 in the tumor tissues of NSCLC patients

First, the mRNA expression level of CXCL13 in lung cancer was analyzed online by the Gene Expression Profiling Interactive Analysis (GEPIA) tool. By analyzing The Cancer Genome Atlas (TCGA) data using log2 (tags per million + 1) for log scale, we found that CXCL13 expression was significantly higher in lung adenocarcinoma (LUAD) and lung squamous cell carcinoma (LUSC) than that in normal lung tissues (Figure 1A). To confirm if the CXCL13 level is elevated in NSCLC patients, we evaluated the CXCL13 expression in two NSCLC tissue arrays—NSC157 and LC20813b—each containing 150 or 192 cases of NSCLC, respectively. Tissue arrays stained by IHC indicated that about 70% of samples from the two arrays were CXCL13 positive, and over 40% of them showed moderate to high levels of CXCL13 expression (Figure 1B). Representative IHC images showed different intensities of CXCL13 expression in normal lung tissues and NSCLC tissues (Figure 1C). Furthermore, we quantified the level of CXCL13 in the plasma samples of NSCLC patients (n = 95) and healthy donors (n = 34) using enzyme-linked immunosorbent assay (ELISA). In line with the previous report,[24] there was an 8-fold increase in the median level (14-fold in mean) of plasma CXCL13 in NSCLC patients compared with the healthy controls (Figure 1D).

Expression profiles of chemokine receptors in T cells

To investigate the expression profiles of chemokine receptors in T cells, the expressions of C-C chemokine receptor type 2b (CCR2b), CXCR3, CXCR4, and CXCR5 were analyzed by flow cytometry. We found that CCR2b, CXCR3, and CXCR4 were expressed in more than 20% of resting T cells. After T cell activation by CD3/CD28 Dynabeads (beads:T cell = 1:1) for 2 days, CCR2 and

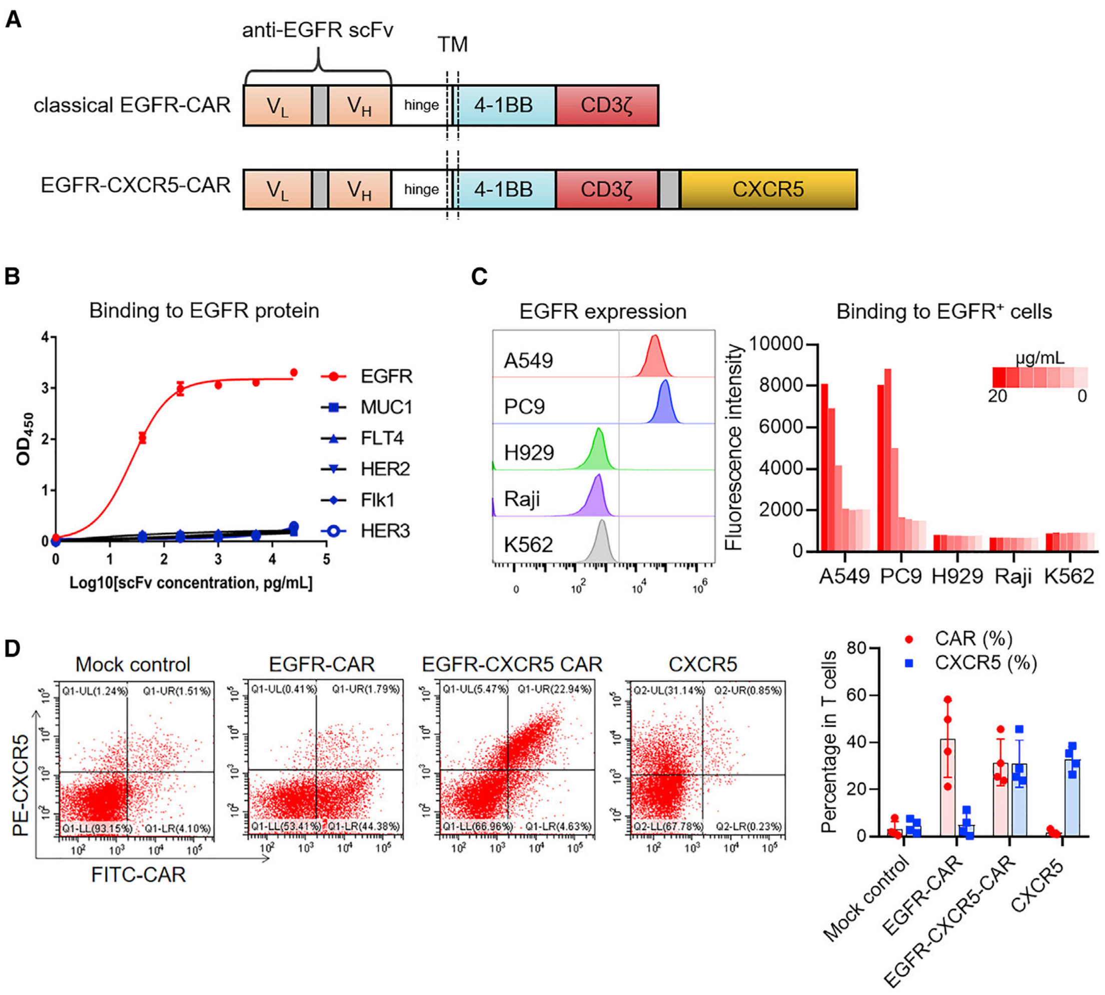

Figure 2. The specificity of EGFR recognition by EGFR scFv and the construction of EGFR-CXCR5 chimeric antigen receptor (CAR)-Ts

(A) Graphical representation of the CAR designed using the anti-EGFR scFv, CD8a hinge, and transmembrane domain, 4-1BB and CD3zeta endodomain. EGFR-CXCR5 was constructed with an additional CXCR5 sequence after the CD3zeta endodomain. (B) ELISA of anti-EGFR scFv with recombinant human immunoglobulin G1 (IgG1) Fc-conjugated EGFR (ErbB1), HER2 (ErbB2), HER3 (ErbB3), MUC1, Flk1 (VEGFR2), and FLT4 (VEGFR3). Recombinant proteins were coated in the plate wells at 5 μg/mL. Anti-EGFR scFv concentration started from 5,000 pg/mL and was diluted 5-fold repeatedly until 8 pg/mL. (C) FACs analysis of A549 and PC9 (LUAD cell lines), H929 (myeloma cell line), Raji (human Burkitt's lymphoma cell line), and K562 (human myelogenous leukemia cell line) stained with anti-EGFR scFv. Concentration started from 20,000 ng/mL and was diluted 10-fold repeatedly until 0.2 ng/mL. (D) The expression of transgenes in lentivirus-transduced T cells was analyzed by flow cytometry using protein L and anti-CXCR5 antibody. Single dot represents individual sample. Error bars represent mean ± SD for each T cell population (n = 4).

CXCR4 expression increased about 2-fold, and CCR3 and CXCR5 expression did not change. Noticeably, the expression of CXCR5 was restricted in CD4 T cells in both resting and activated T cells (Figure 1E). This is consistent in that CXCR5 is constitutively expressed in a small group of CD4 T_{fh} cells that plays a critical role in mediating the selection and survival of B cells in germinal centers. In addition, the mRNA expression levels of chemokines (CCL2, CCL7, CCL8, CCL13, CXCL4, CXCL9, CXCL10, CXCL11, CXCL12, and CXCL13) corresponding to the four chemokine receptors in various cancers were analyzed using the online Oncomine database (threshold for fold change [FC] > 2, p < 0.05, and rank = top 10), and the results showed that only CXCL13 (unique ligand for CXCR5) expression was elevated in lung cancer (Figure S1). Thus, the chemotactic signaling between CXCR5 and its ligand CXCL13 may be assembled into CAR-T cells to enhance their antitumor activity against solid tumors.

T cells expressing a classical EGFR-targeting CAR and the CXCR5 receptor

Based on these findings, we engineered a classical second generation anti-EGFR-CAR construct using the variable domain of cetuximab (named anti-EGFR single-chain fragment variable [scFv]) with a chemokine receptor CXCR5 to facilitate CAR-T migration to CXCL13-positive tumors (Figure 2A). Specificity of the anti-EGFR scFv was

validated in detail at both molecular and cellular levels. ELISA-based assays were used to confirm that the anti-EGFR scFv binds only with the EGFR protein in a dose-dependent manner. No cross-reactivity with other EGFR family proteins (HER2, HER3), vascular endothelial growth factor receptor (VEGFR) family proteins (VEGFR2, VEGFR3), and MUC1 protein was observed (Figure 2B). The anti-EGFR scFv could also label EGFR-positive cell lines (A549 and PC9) but not EGFR-negative cell lines (Figure 2C). Different from the conventional EGFR-CAR, transduction with the EGFR-CXCR5-CAR in primary human T cells yielded higher proportion of CXCR5 and CAR dual-positive T cells (Figure 2D).

EGFR-CXCR5-CAR showed similar cytotoxicity as classic EGFR-CAR but enhanced migration *in vitro*

To evaluate the *in vitro* cytotoxic efficacies of CAR-T cells against NSCLC tumor cell lines, we first used a real-time cytotoxicity assay based on IncuCyte. It established that EGFR-CXCR5-CAR-T cells eradicated A549 and PC9 cells within 48 h but not EGFR-negative cell line K562 (Figures 3A and 3B). The released cytokine profile was also measured during the cell killing. High levels of interferon (IFN)-γ and interleukin (IL)-2 were recorded 20 h after CAR-T cells, and PC9 tumor cells were co-cultured. There were no significant differences between cytokines released by EGFR-CAR and that by EGFR-CXCR5-CAR, suggesting that the ectopic expression of CXCR5 does not affect T cell cytotoxicity function (Figure 3C). Next, we tested whether CXCR5 expression improved T cell migration. For the *in vitro* Transwell assay, we loaded CAR-T cells in the upper chamber and allowed them to migrate to the lower chamber under CXCL13 gradient. After 4, 8, and 16 h, the number of migrated T cells in the lower chamber was estimated by flow cytometry using CountBright Absolute Counting Beads. Compared with the classical EGFR-CAR-T cells, EGFR-CXCR5-CAR-T cells showed that a significantly larger number of cells migrated to the bottom (Figure 3D). Consistent with the aid of chemotaxis, migration of CAR-T cells was positively correlated with CXCL13 concentration, whereas high concentrations of CXCL3 did not promote CAR-T cell proliferation (Figure 3E), suggesting that the increase in cell number was mediated by chemotaxis.

EGFR-CXCR-CAR demonstrated increased migration to CXCL13-positive tumors *in vivo*

To confirm that the addition of CXCR5 facilitates T cell migration to CXCL13-positive tumors *in vivo*, A549 cells were transfected with human CXCL13-expressing vector to generate the A549-CXCL13 cell line, and the EGFR expression and CXCL13 release were confirmed (Figures 4A and 4B). EGFR-CXCR5-CAR-T cells, EGFR-CAR-T cells, and untransduced mock control T cells were all labeled with ^{89}Zr-oxine, and the proliferative activity was detected at 37°C for 7 days. The results showed that ^{89}Zr-oxine labeling did not affect the proliferation of CAR-T cells (Figure 4C). Subcutaneous injection of A549 cells in nude mice was widely used for both basic research and drug discovery, including CAR-T therapy. Here, we inoculated the non-obese diabetic scid gamma (NSG) mice with A549 cells at the left and A519-CXCL13 cells at the right flank. After 14 days of tumor establishment, ^{89}Zr-EGFR-CXCR5-CAR-T cells, ^{89}Zr-EGFR-CAR-T cells, and ^{89}Zr-mock control T cells (n = 3 per group) were injected, respectively, via tail vein, and T cell distribution was monitored at 2, 24, 72, and 168 h post-injection. Intense isotope signals were recorded in the lung, followed by liver and spleen, 2 h after T cell infusion (Figures 4D and S2). We further analyzed the radioactivity uptake values of the A549 tumor region on the left (green circle) and the A549-CXCL13 tumor region on the right (red circle). The ^{89}Zr-EGFR-CXCR5-CAR-T cells showed trafficking to the A549-CXCL13 tumor 72 h post-infusion, whereas the ^{89}Zr-EGFR-CAR-T cells and ^{89}Zr-mock control T cells were barely detectable (Figure 4D). EGFR-CXCR5-CAR-T cells accumulated in the A549-CXCL13 tumor at a faster rate over time, whereas the signal from EGFR-CAR-T cells remained at a lower level at 168 h post-infusion. Quantification of the radio-isotope signal from each group confirmed that CXCR5-redirected CAR-T cells showed the highest intensity at A549-CXCL13-positive sites, about 2-fold higher at 72 h and 3-fold higher at 168 h compared with classical EGFR-CAR (Figure 4E).

Antitumor capacity of EGFR-CXCR5-CAR-T cells *in vivo*

We next tested the ability of each type of activated T cells to exert antitumor activity *in vivo*. A549 cells were inoculated at the left side, and A549-CXCL13 cells were inoculated at the right side. 5×10^5 CAR-positive T cells per mice were infused via tail vein 10 days post-tumor inoculation. We observed that EGFR-CXCR5-CAR-T almost completely eliminated the A549-CXCL13 tumor at the right side (Figure 5A), whereas there was no significant effect on the A549 tumor on the left, suggesting that EGFR-CXCR5-CAR-T tended to migrate to kill CXCL13-positive tumors. EGFR-CAR-T showed no preference to the tumors at the left or right and only partially inhibited tumor growth at both sides, which were supported by both the tumor isotope imaging and volume measurements. We then analyzed the tumor volumes on the left (A549) and right (A549-CXCL13) sides and found that EGFR-CXCR5-CAR-T was more effective in eradicating A549-CXCL13 (right-side tumor) than classical EGFR-CAR-T (Figure 5B). We further analyzed the copy number of the CAR gene in the left and right tumor tissues and found a higher CAR copy number in the right A549-CXCL13 tumor (Figure 5C). These results confirm our hypothesis that the addition of CXCR5 facilitates T cell migration and enhances killing of CXCL13-positive tumors *in vivo* (Figure 6).

DISCUSSION

In this study, prompted by the observation of the CXCL13 overexpression on certain NSCLC patients, we implemented the strategy to co-express CXCR5 with the anti-EGFR-CAR-T to facilitate T cell migration to the tumor site. Both *in vitro* and *in vivo* experiments confirmed the original hypothesis that the overexpression of CXCR5 improves the migration of CAR-T cells to the CXCL13-positive tumor site, as shown directly by tracking CAR-T cells *in vivo*. The increased migration contributed to the enhanced CAR-T cytotoxicity on EGFR-CXCL13-positive tumor cells. The results from this study warrant the product to be tested in a carefully designed clinical trial. To benefit from this design, patients need to be EGFR and CXCL13 double positive, which can be readily confirmed by IHC comparing tumor and adjacent tissues.

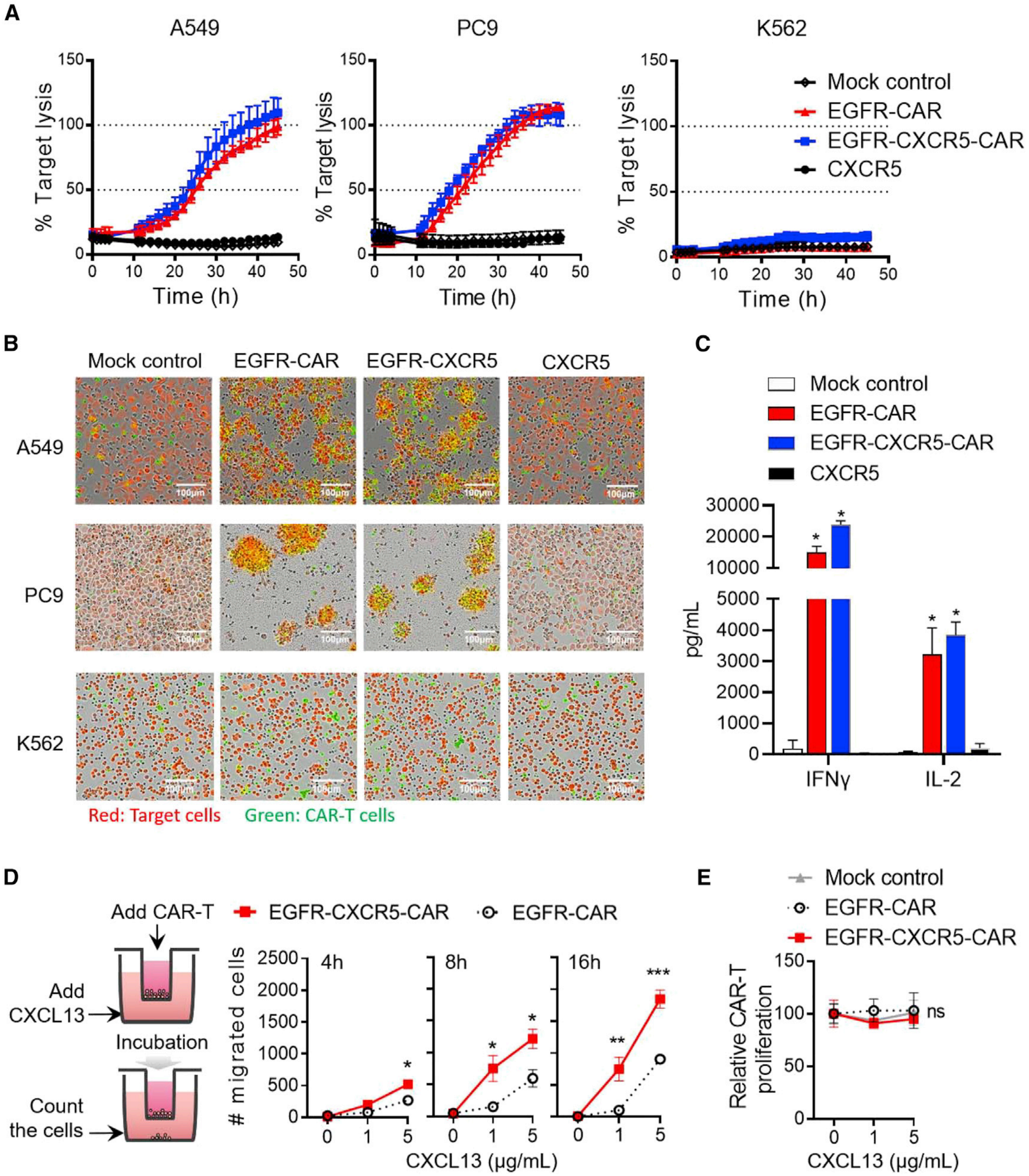

Figure 3. Evaluation of the antitumor efficacy and chemotaxis migration of EGFR-CXCR5-CAR-T cells *in vitro*

(A) Analysis of the cytotoxicity of EGFR-CXCR5-CAR-T cells against PC9, A549, and K562 cells. Tumor cell killing was measured via an IncuCyte assay over 48 h. SYTOX Green and CellTrace Far Red double-positive tumor cells (yellow) were calculated. Error bars represent mean ± SD for each time point. (B) Real-time cell killing image. Target cells were red, and CAR-T cells were green. (C) Cytokine production by CAR-T cells co-cultured with PC9 tumor cells. CAR-T cells were co-cultured 10:1 with tumor cells in 96-well plates for 20 h. Levels of IFN-γ and IL-2 in supernatant were determined by ELISA. Error bars represent mean ± SD for each group. (D) Chemotaxis migration of CAR-Ts toward various concentrations of recombinant human CXCL13 at different time courses of 4 h, 8 h, and 16 h. Error bars represent mean ± SD for each group (n = 3). *$p < 0.05$ derived via unpaired t test. (E) CAR-T cell proliferation assay with indicated CAR-T cells cocultured with various concentrations of recombinant human CXCL13.

EGFR is one of the validated targets for NSCLC treatment. However, the clinical application of EGFR ectodomain-targeted therapy has been limited by the prevalence of EGFR in normal tissues, such as skin and endothelium of blood vessels, which raises the concern for on-target, off-tumor toxicity,[26] including oral mucositis, oral ulcer, gastrointestinal hemorrhage, desquamation, and pruritus. Acute respiratory distress syndrome (displayed an acute pulmonary edema) has been reported in individual patients receiving EGFR-CAR-T

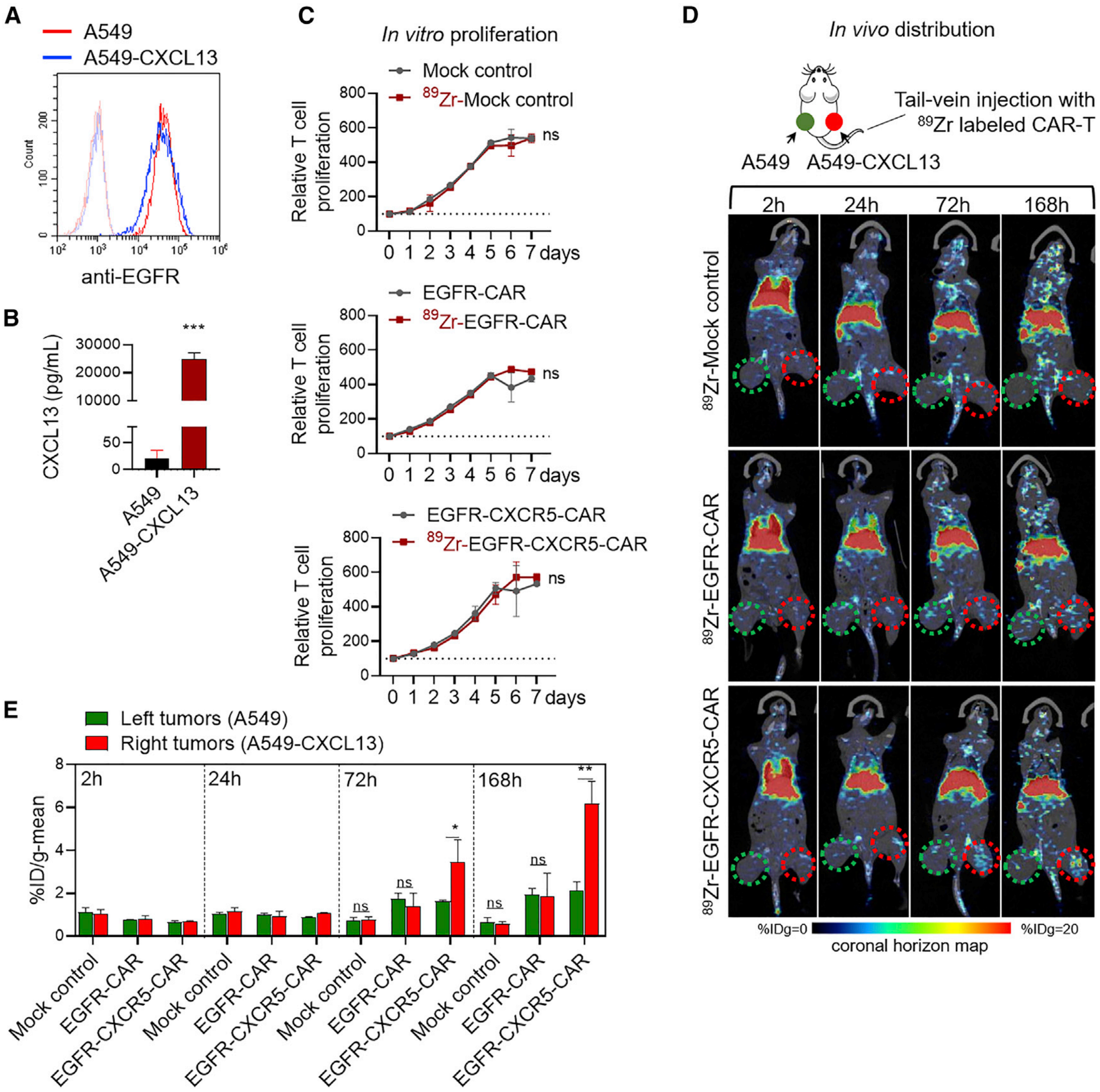

Figure 4. *In vivo* tracking of the migration of ^{89}Zr-oxine-labeled CAR-T to A549 and A549-CXCL13 tumors using micro-PET/CT scan

(A) EGFR expression in the A549 cell line stably expressing the CXCL13 gene (A549-CXCL13) after lentiviral transduction and selection. (B) Increased secretion of CXCL13 generated by A549-CXCL13 cells. ***$p < 0.001$. (C) The effects of ^{89}Zr-oxine labeling on T cell proliferation. (D) Whole-body PET imaging, quantitative PET analysis, and biodistribution of ^{89}Zr-labeled T cells in tumor-bearing mice. ^{89}Zr-labeled mock T cells, ^{89}Zr-EGFR-CAR-T cells, or ^{89}Zr-EGFR-CXCR5-CAR-T cells were tail-vein injected into NSG mice inoculated with A549 tumor cells at the left and A549-CXCL13 tumor cells at the right side. Isotopic distribution of ^{89}Zr was quantified and plotted in a coronal horizon map at different time points of 2, 24, 72, and 168 h post-injection. The red and green circles represent the A549 tumor region and A549-CXCL13 tumor region, respectively. (E) Accumulated isotope signaling in A549 tumor region (green circle) and A549-CXCL13 tumor region (red circle). The percentage injection dose rate ([%ID]/g value) was calculated. Error bars represent mean ± SD for each group (n = 3).

therapy.[27] Notably, many CAR-Ts targeting tumor-associated antigens may have potential on-target/off-tumor toxicities, such as HER2, GD2, Claudin18.2, etc. More optimized CAR structural designs need to be developed to enable CAR-T to be widely used in the treatment of solid tumors. In this study, the co-expression of CXCR5 in CAR-T was designed to not only increase the infiltration of CAR-T cells but also to mitigate potential off-tumor toxicity, due to our results that CAR-T only infiltrates into EGFR and CXCL13 double-positive tumor sites and produces a killing effect.

For T cell infiltration, the strategy of guiding T cell migration by chemotaxis may be more appealing over the local delivery approach. For example, direct intra-tumor injection of CAR-T cells has been studied for some tumor types, such as IL-13 receptor (IL-13R)α CAR-T for glioblastoma. Although transient local tumor shrinkage was observed, this approach becomes unrealistic for the late-stage cancer patient with multiple metastatic lesions. Therefore, taking advantage of cell intrinsic mechanisms to increase T cell infiltration is probably more efficient. Chemotaxis has been successfully utilized previously to increase

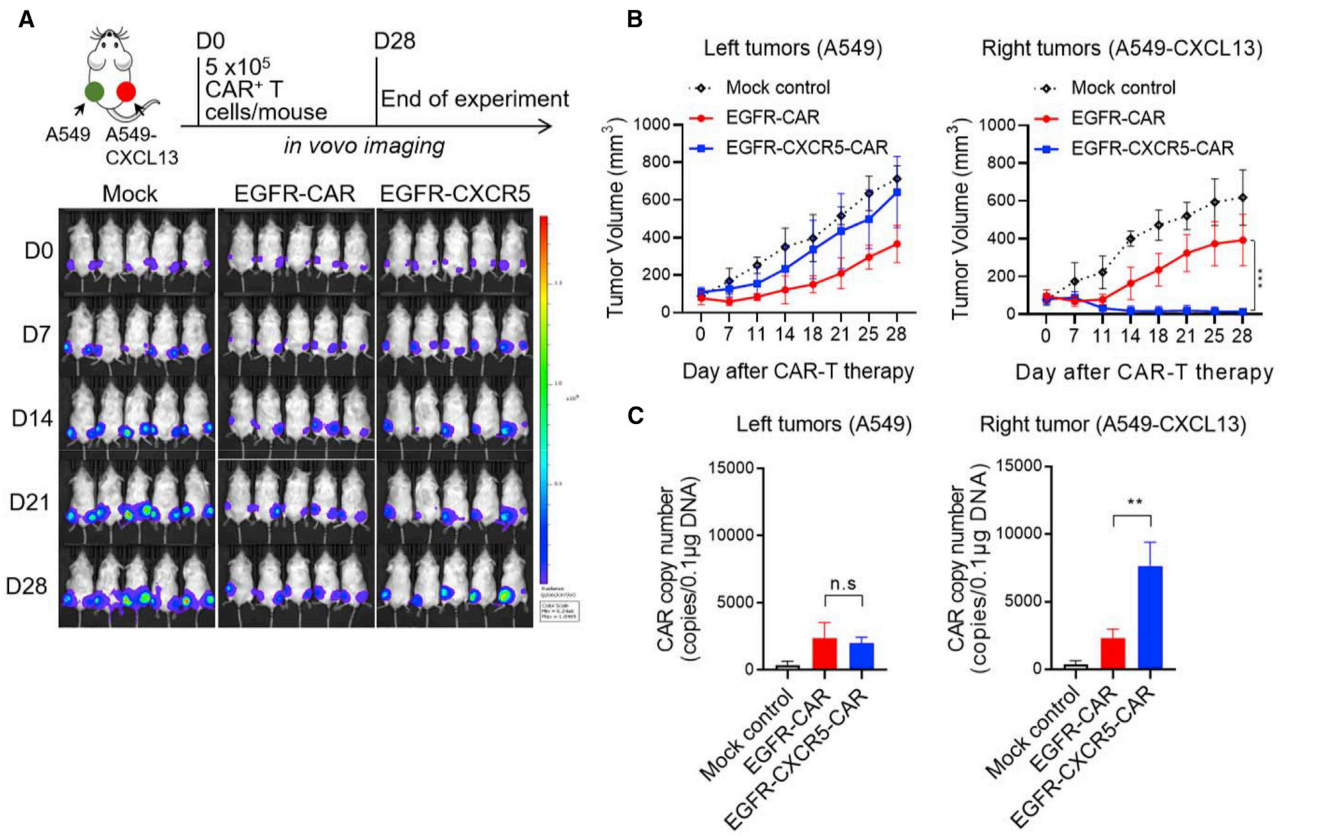

Figure 5. Antitumor efficacy of CAR-T cells *in vivo*

(A) Serial bioluminescence imaging of NSG mice injected subcutaneously with $A549^{luc}$ cells on the left flank and $A549^{luc}$-CXCL13 cells on the right flank. 10 days after tumor engraftment, the mice were injected with 5×10^5 CAR^+ T cells as indicated. n = 5 mice per group. Error bars represent mean ± SD for each time point (n = 5). (B) The tumor volume of the left tumors ($A549^{luc}$) and right tumors ($A549^{luc}$-CXCL13) over 28 days was measured. Error bars represent mean ± SD for each time point (n = 5). (C) The copy number of CAR gene in the left and right tumor tissues was analyzed. **$p < 0.01$.

CAR-T migration *in vivo*. For example, overexpression of CCR4 by CD30-CAR-T cells enhanced migration in response to Hodgkin's lymphoma secreting its ligand CCL17.[28] Craddock et al.[29] demonstrated enhanced tumor-tactic migration of GD2-specific CAR-T cells by expressing the CCR2b. A functional CCR2 receptor enhanced tumor localization and tumor eradication by retargeted human T cells expressing a mesothelin-specific CAR.[30] During chronic HIV infection, replication is concentrated in T_{fh} cells located within B cell follicles. Accordingly, an anti-simian immunodeficiency virus (SIV) CAR with CXCR5 expression has been constructed to provide long-term durable remission (functional cure) of HIV and SIV infections.[31] In addition, a recent study also identified that CCL19, CCL20, and CXCL13 are highly expressed in lung cancer, and subsequent co-expression of CCL20 receptor CCR6 in CAR-T increased cell migration and tumor killing.[32]

As CXCR5 is an endogenous B cell homing receptor responding to the expression of CXCL13 in B cell zones, the same strategy that we implemented here could be potentially applied to the treatment of B cell malignancies. Currently, we are evaluating the potential advantage of CXCR5/CD19 CAR-T over the classical CD19 CAR-T in treating B cell lymphomas. Moreover, CXCL13-CXCR5 co-expression regulates epithelial-to-mesenchymal transition of tumor cells during lymph node metastasis.[33] It would be interesting to check whether this design could also help prevent cancer metastasis.

Our current study focused on the addition of a tumor-targeting chemokine receptor to improve T cell migration and recruitment. More broadly, other challenges, such as the immunosuppressive tumor microenvironment (TME) in solid tumors that leads to the inactivation and exhaustion of effector T cells, still remain to be overcome. To further improve the clinical outcome of chemokine receptor-directed CAR-T therapy on solid tumors, combinations with other therapies such as anti-programmed cell death protein 1 (PD-1)/programmed cell death ligand 1 (PD-L1) or oncolytic virus treatment are worth exploring in efforts to develop multi-modality approaches to cancer treatment.

MATERIALS AND METHODS

Patient samples

The study was approved by the Research Ethics Committees of all participating sites. Human plasma samples were obtained through

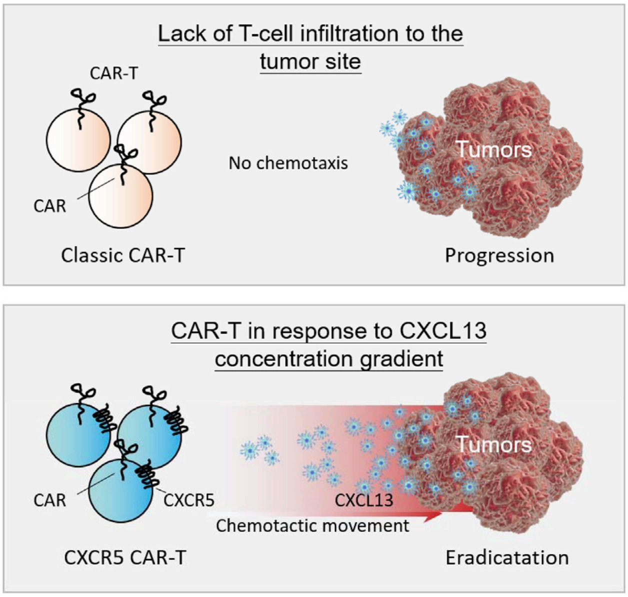

Figure 6. Addition of CXCR5 facilitates T cell migration

The chemokine CXCL13 is highly expressed in various tumors including lung carcinoma, whereas the classical CAR-T does not effectively infiltrate into tumor regions due to the absence of CXCR5 receptor expression. Chemotactic movement is a taxis in response to a chemical concentration gradient. When CAR-T cells are modified with the CXCR5 receptor, the motorized CAR-T cells could infiltrate into the tumor site along the gradient of CXCL13 to further clear the tumor cells.

informed consent by Central Hospital of Panyu District, Guangzhou, Guangdong, China.

CXCL13 expression measurement

The CXCL13 level in the blood plasma was quantified by the CXCL13 ELISA kit from OriGene (catalog number [cat. no.] EA800062) according to the manufacturer's instructions. The protein level in the tissue sample was determined by IHC. Two middle-advanced-stage lung cancer tissue microarrays (NSC157 and LC20813b), each containing 150 and 192 cases, respectively, were purchased from Alena-Bio (Xi'an, China). Briefly, formalin-fixed paraffin-embedded (FFPE) tissue array slides were deparaffinized with xylene and rehydrated in serial dilutions of ethanol. Antigens were retrieved in the citrate antigen repair buffer (pH 6.0; Wuhan Servicebio Technology), heated in a steam pressure cooker, followed by washing in PBS (pH 7.4) three times. To block endogenous peroxidases, tissue array slides were covered with 3% hydrogen peroxide and incubated at room temperature for 25 min in the dark. Slides were further blocked with rabbit serum for 30 min at room temperature. Primary polyclonal goat anti-human CXCL13 (cat. no. AF801, 1:20; R&D Systems) antibodies were applied and incubated in a humidified box overnight at 4°C. The slides were washed in PBS (pH 7.4) three times, and 0.5 μg/mL horseradish peroxidase-conjugated rabbit anti-goat secondary antibody (Invitrogen) was applied for 50 min at room temperature. After washing in PBS (pH 7.4), slides were treated with 3,3′-diaminobenzidine (DAB)-peroxidase substrate solution (Wuhan Servicebio Technology) and counter stained with hematoxylin. The immunoreactivity score (IRS) was calculated as IRS (0–12) = CP (0–4) · SI (0–3), where CP is the percentage of CXCL13-positive tumor cells, and SI is the staining intensity. The CP was assessed as follows: 0 (<1% positive tumor cells), 1 (1%–25% positive tumor cells), 2 (26%–50% positive tumor cells), 3 (51%–75% positive tumor cells), and 4 (>75% positive tumor cells). The SI was evaluated on the basis of the following criteria: 0 (no staining), 1 (weak staining = light yellow), 2 (moderate staining = yellow), and 3 (strong staining = yellow brown). Eventually, the IRS ranging from 0 to 12 was obtained, and the final IRS was defined as 0 for negative expression, 1−4 for low expression, 5−8 for medium expression, and 9−12 for high expression.

Flow cytometry

The monoclonal antibodies (mAbs) used to examine the expression of indicated proteins were purchased from the following vendors: (1) BioLegend (London, UK): fluorescein isothiocyanate (FITC)-mouse anti-human CCR2 (cat. 357216), allophycocyanin (APC)-Cyanine7 (Cy7)-mouse anti-human CXCR5 (cat. 356926), phycoerythrin (PE)-mouse anti-human-CXCR5 (cat. 356904), and PE-conjugated anti-human EGFR (cat. 352904). (2) BD Biosciences (San Jose, CA, USA): PerCP-Cy5.5-mouse anti-human CXCR3 (cat. 560832) and PE-Cy7-mouse anti-human CXCR4 (cat. 560669). (3) ACRO Biosystems: FITC-labeled recombinant protein L (cat. RPL-PF141). Protein L was used to detect the cell surface expression of CARs because it selectively binds to variable light chains (kappa chain) of immunoglobulin without interfering with antigen-binding property of the antibodies. Data were acquired on a CytoFLEX Flow Cytometer (Beckman Coulter) and analyzed by CytExpert software or FlowJo version 7.6.1 (Tree Star).

Antigen antibody-binding assay

EGFR scFv fused with a six-histidine (6× His) tag was expressed in HEK293T cells. Recombinant human HER3, VEGFR2 (Flk1), and VEGFR3 (Flt4) were purchased from Sino Biological. Recombinant human EGFR, MUC1, and HER2 were obtained from ACRO Biosystems. For ELISA assay, all six recombinant proteins were coated into a 96-well plate (0.5 μg/well) in 100 μL of coating buffer overnight, and then different concentrations (8, 40, 200, 1,000, and 5,000 pg/mL) of EGFR scFv (100 μL/well) were added. After incubation of horseradish peroxidase (HRP)-conjugated mouse anti-His antibody (0.025 μg/well; BioLegend) and 3,3′,5,5′-tetramethylbenzidine (TMB) substrate, the optical density (OD) value at 450 nm was determined using Tecan Infinite F50. In addition, rabbit anti-human MUC1, HER2, HER3, VEGFR2, and VEGFR3 antibodies (Sino Biological) were used as positive controls for detecting recombinant human MUC1, HER2, HER3, VEGFR2, and VEGFR3 proteins. The experiments were repeated four times independently.

For fluorescence-activated cell sorting (FACS) assay, 0.5×10^6 A549, PC9, H929, Raji, and K562 cells were mixed with EGFR scFv (0.2−20,000 pg/mL) and incubated at 4°C for 1 h. After washing and centrifugation, cells were stained with 0.6 μg/mL PE-conjugated

mouse anti-His (cat. J095G46; BioLegend, London, UK). Data were collected by a CytoFLEX Flow Cytometer (Beckman Coulter), and the fluorescence intensity in each sample was analyzed. Experiments were repeated three times independently.

CAR design and synthesis

EGFR scFv was derived from the EGFR mAb cetuximab but with a point mutation at heavy chain variable region N88Q to avoid possible glycosylation. The sequence was codon optimized for expression in human cells, synthesized by Guangzhou IGE Biotechnology, and then introduced into a pCDH lentiviral expression vector containing a CD8α hinge transmembrane domain, a CD137 (4-1BB) co-stimulatory motif, and a CD3ζ signaling domain. For EGFR-CXCR5-CAR, the EGFR-CAR was inserted into the full length of CXCR5 (372 amino acids [aa]) at the C-terminal separated by T2A. The full-length CXCR5 contains its native secretory signal. The resultant CAR constructs were sequence verified and used for downstream applications.

Production of lentiviral vector and CAR-T cell transduction

Lentiviral vector supernatant for the EGFR-CAR or EGFR-CXCR5-CAR was produced by transient transfection of adherent 293T cells (Takada) with the corresponding CAR plasmid and three packaging plasmids: pLP1, pLP2, and pLP/vesicular stomatitis virus glycoprotein. The medium was exchanged 4 h after transfection. After an additional 48 h, the cell supernatant was pooled and filtered with a 0.45-μm filter, followed by Benzonase treatment (Merck) for 16 h. Then, the harvest was passed through a Mustang Q ion-exchange capsule (Pall, Ann Arbor, MI, USA). The Mustang Q membrane was washed using 50 mM Tris-HCl (pH 8.0) with 750 mM NaCl and then eluted in fractions using 50 mM Tris-HCl (pH 8.0) with 1.5 M NaCl and diluted with phosphate buffer (pH 7.2). The elution was further concentrated approximately 10-fold by a 300-kDa tangential flow filtration (TFF) column. The final concentrate was formulated with human serum albumin (HSA) to 2%, filtered with a 0.22-μm filter, aliquoted to 2 mL cryotubes, quick frozen on dry ice, and stored at −80°C.

CAR-T cells were generated from commercial normal donor peripheral blood mononuclear cells (PBMCs) from AllCells. The cells were further enriched from PBMC by CD3 magnetic beads (Miltenyi) and stimulated by anti-CD3/CD28 beads (Dynabeads, human T activator CD3/CD28; Life Technologies) at a 1:3 bead:T cell ratio and then cultured in H3 medium (Takara) with 4% human AB serum and 10 ng/mL recombinant human IL-7 and IL-15 (Miltenyi). Cells were exposed to lentivirus containing supernatant on days 2 and 3 with MOI = 5 on retronectin-coated non-tissue culture plates (Takara/Clontech). Beads were magnetically removed on day 4 or 5, and cells were further expanded for 3−5 days in H3 media containing 10 ng/mL recombinant human IL-7 and IL-15 until use *in vitro* or *in vivo*.

Cell lines

The human NSCLC cells A549, $A549^{luc}$ (luciferase-expressing A549), and PC-9, chronic myelogenous leukemia (CML) cell-line K562; myeloma cell-line H929; and Burkitt's lymphoma cell line Raji were obtained from Shanghai Zhong Qiao Xin Zhou Biotechnology and Procell Life Science & Technology. To generate CXCL13-expressing $A549^{luc}$ cells ($A549^{luc}$-CXCL13), cDNA, encoding the full-length CXCL13 (aa 1−109), was polymerase chain reaction (PCR) amplified and directionally cloned into the lentiviral vector pCDH-Cytomegalovirus-multiple cloning site-elongation factor 1-puromycin by incorporating the restriction enzyme sites BamHI and NotI into the PCR forward and reverse primers, respectively. The supernatant from the transfected HEK293 T cells was used to infect $A549^{luc}$ cells, and the infected cells were selected with 1 mg/mL puromycin (Sangon Biotech, Shanghai). A549 and $A549^{luc}$ cells were maintained in DMEM supplemented with 10% fetal bovine serum (FBS). $A549^{luc}$-CXCL13 was maintained in DMEM supplemented with 10% FBS and 1 mg/mL puromycin. PC-9, Raji, and H929 cells were maintained in RPMI-1640 medium supplemented with 10% FBS. K569 cells were cultured in Iscove's modified Dulbecco's medium (IMDM) supplemented with 10% FBS. All cell culture medium was supplemented with 10 mM HEPES; 2 mM GlutaMAX; and 100 units/mL penicillin, 100 μg/mL streptomycin, and 0.25 μg/mL amphotericin B.

Cytotoxicity assay

Killing assays were performed by co-culturing 50,000 CAR^+ T cells with 50,000 CellTrace Far Red-labeled tumor cells in complete media in a 96-well plate with SYTOX Green as an indicator of dead cells. Images were acquired every 2 h using an IncuCyte (Sartorius). The percentage of tumor cells killed was calculated by dividing the number of CellTrace Far Red and SYTOX Green double-positive tumor cells by the total number of CellTrace Far Red-positive tumor cells at every time point.

Cytokine measurements

Human IFN-γ and IL-2 from a cell culture supernatant were measured by an ELISA development kit (4A Biotech, Beijing) and used according to the manufacturer's instructions. The CXCL13 level in A549 and A549-CXCL13 cells was quantified by a CXCL13 ELISA kit (cat. no. EA800062; OriGene). Cytokine concentrations were calculated by standard curve regression.

In vitro Transwell assay

Transduced EGFR-CAR-T and EGFR-CXCR5-CAR-T cells were starved overnight in serum-free medium before chemotaxis assay. 3×10^5 cells/well were placed in the upper chamber of a 6.5-mm diameter polycarbonate Transwell membrane in 24-well culture plates (5 μm pore size; Costar Transwell, Corning, NY, USA), and lower chambers contained 600 μL serum-free medium with different concentrations of CXCL13. Serum-free medium with no added chemokine served as a negative control. CAR-T cells in the upper chamber were allowed to migrate for 16 h at 37°C and 5% CO_2. Migrated cells were collected from the lower compartment. CountBright Absolute Counting Beads (Invitrogen) were added, and then cells were resuspended in a fixed volume. Data were collected using a Beckman Coulter CytoFLEX flow cytometer and analyzed with FlowJo software (Tree Star).

Isotope labeling and biodistribution of CAR-T cells *in vivo*

^{89}Zr labeling and biodistribution of CAR-T cells were performed by MITRO Biotech (Nanjing, China). In brief, 3 × 10^7 ^{89}Zr-labeled mock T cells, ^{89}Zr-EGFR-CAR-T cells, or ^{89}Zr-EGFR-CXCR5-CAR-T cells were tail-vein injected into NSG mice inoculated with A549 tumor cells at the left and A549-CXCL13 tumor cells at the right side. The CAR-T cell bio-distribution was measured at different time points of 2, 24, 72, and 168 h post-injection. After positron emission tomography (PET)/computed tomography (CT) scanning, image reconstruction was carried out. PMOD software was used to process the images and data, and the brain, heart, liver, spleen, lung, kidney, tibia, bone and joint, muscle, and tumor organs were mapped as regions of interest. The radioactivity concentration (i.e., the radioactivity value per unit volume) of the region of interest was obtained. Then, the activity at each time point was decayed and corrected. The percentage injection dose rate per gram ([%ID/g] value) of tissue of each organ was calculated according to the dose of administration.

Quantitative PCR (qPCR)

For detection of copy number of the CAR gene in tumors, genomic DNA was extracted using the DNeasy Blood & Tissue kit (QIAGEN, Germantown, MD, USA) from tumor tissues of mice. A pair of CAR-specific Scorpion primers and probes (TaqMan TAMRA from Applied Biosystems, Thermo Fisher Scientific) was used to perform qPCR on an ABI 7500 PCR system, along with 100 ng of genomic DNA. Standard curves were prepared using serial dilutions of the EGFR-CAR plasmid. The copy number of unknown samples was interpolated from the standard curves using GraphPad Prism software (La Jolla, CA, USA).

Mice

NSG mice were purchased from the Nanjing Model Animal Research Center and anesthetized with 3% isoflurane (Minrad International, Buffalo, NY, USA) in an incubation chamber. Anesthesia on the stereotactic frame (David Kopf Instruments, Tujunga, CA, USA) was maintained at 2% isoflurane delivered through a nose adaptor. 5 × 10^6 A549^{luc} and A549^{luc}-CXCL13 cells were injected into the left and right side, respectively, of 6-to 10-week-old NSG mice using a blunt-end needle (75N, 26s gauge, 5 μL; Hamilton, Reno, NV, USA). Tumor formation was followed by bioluminescence imaging on an IVIS Spectrum instrument (Caliper Life Sciences) and quantified with Living Image software (PerkinElmer, Waltham, MA, USA). 10 days after tumor implantation and after confirmation of tumor formation by bioluminescence, mice were randomized and treated with 5 × 10^5 CAR$^+$ T cells or an equivalent number of T cells (matched for total T cell dose) intravenously by tail-vein injection. Isoflurane-anesthetized animals were imaged using the IVIS system (Caliper Life Sciences) 10 min after 3 mg D-luciferin (PerkinElmer) was injected intraperitoneally. Living Image (PerkinElmer, Waltham, MA, USA) software was used to analyze the IVIS data.

Statistical analysis

All cytometric assays were analyzed using FlowJo software (Tree Star, USA). All standard curve regression analyses were performed by GraphPad Prism (GraphPad Software, La Jolla, CA, USA). Data are presented as mean ± SD, unless otherwise stated. Statistical comparisons between groups were performed using the unpaired two-tailed Student's t test to calculate p value unless otherwise stated. Statistical significance was defined as $p < 0.05$.

SUPPLEMENTAL INFORMATION

Supplemental information can be found online at https://doi.org/10.1016/j.omto.2021.07.003.

ACKNOWLEDGMENTS

The authors would like to acknowledge Nanjing Mitro, Inc., for helping to set up radio-isotope labeling of CAR-T cells. Work was funded by Guangzhou Bio-gene Co., Ltd., and Pearl River S&T Nova Program of Guangzhou (201906010056) from Guangzhou Municipal Science and Technology Bureau.

AUTHOR CONTRIBUTIONS

W.M., J.G., G.L., and M.L. conceived the study and wrote and revised the manuscript. J.G., G.L., Y.Z., Z.H., and W.D. conducted the majority of experiments. All of the authors contributed to the writing and editing of the manuscript.

DECLARATION OF INTERESTS

G.L., J.G., W.D., W.M., and M.L. are employees of Guangzhou Bio-gene Technology Co., Ltd., for which potential product is studied in this work. The other authors have no competing interests.

REFERENCES

1. Turtle, C.J., Hay, K.A., Hanafi, L.A., Li, D., Cherian, S., Chen, X., Wood, B., Lozanski, A., Byrd, J.C., Heimfeld, S., et al. (2017). Durable Molecular Remissions in Chronic Lymphocytic Leukemia Treated With CD19-Specific Chimeric Antigen Receptor-Modified T Cells After Failure of Ibrutinib. J. Clin. Oncol. *35*, 3010–3020.
2. Kochenderfer, J.N., Somerville, R.P.T., Lu, T., Yang, J.C., Sherry, R.M., Feldman, S.A., McIntyre, L., Bot, A., Rossi, J., Lam, N., and Rosenberg, S.A. (2017). Long-Duration Complete Remissions of Diffuse Large B Cell Lymphoma after Anti-CD19 Chimeric Antigen Receptor T Cell Therapy. Mol. Ther. *25*, 2245–2253.
3. Park, J.H., Rivière, I., Gonen, M., Wang, X., Sénéchal, B., Curran, K.J., Sauter, C., Wang, Y., Santomasso, B., Mead, E., et al. (2018). Long-Term Follow-up of CD19 CAR Therapy in Acute Lymphoblastic Leukemia. N. Engl. J. Med. *378*, 449–459.
4. O'Rourke, D.M., Nasrallah, M.P., Desai, A., Melenhorst, J.J., Mansfield, K., Morrissette, J.J.D., Martinez-Lage, M., Brem, S., Maloney, E., Shen, A., et al. (2017). A single dose of peripherally infused EGFRvIII-directed CAR T cells mediates antigen loss and induces adaptive resistance in patients with recurrent glioblastoma. Sci. Transl. Med. *9*, eaaa0984.
5. Ahmed, N., Brawley, V., Hegde, M., Bielamowicz, K., Kalra, M., Landi, D., Robertson, C., Gray, T.L., Diouf, O., Wakefield, A., et al. (2017). HER2-Specific Chimeric Antigen Receptor-Modified Virus-Specific T Cells for Progressive Glioblastoma: A Phase 1 Dose-Escalation Trial. JAMA Oncol. *3*, 1094–1101.
6. Ahmed, N., Brawley, V.S., Hegde, M., Robertson, C., Ghazi, A., Gerken, C., Liu, E., Dakhova, O., Ashoori, A., Corder, A., et al. (2015). Human Epidermal Growth Factor Receptor 2 (HER2) -Specific Chimeric Antigen Receptor-Modified T Cells for the Immunotherapy of HER2-Positive Sarcoma. J. Clin. Oncol. *33*, 1688–1696.
7. Richards, R.M., Sotillo, E., and Majzner, R.G. (2018). CAR T Cell Therapy for Neuroblastoma. Front. Immunol. *9*, 2380.
8. Zhou, R., Yazdanifar, M., Roy, L.D., Whilding, L.M., Gavrill, A., Maher, J., and Mukherjee, P. (2019). CAR T Cells Targeting the Tumor MUC1 Glycoprotein Reduce Triple-Negative Breast Cancer Growth. Front. Immunol. *10*, 1149.

9. Watanabe, K., Luo, Y., Da, T., Guedan, S., Ruella, M., Scholler, J., Keith, B., Young, R.M., Engels, B., Sorsa, S., et al. (2018). Pancreatic cancer therapy with combined mesothelin-redirected chimeric antigen receptor T cells and cytokine-armed oncolytic adenoviruses. JCI Insight *3*, 99573.

10. Li, H., Huang, Y., Jiang, D.-Q., Cui, L.-Z., He, Z., Wang, C., Zhang, Z.-W., Zhu, H.-L., Ding, Y.-M., Li, L.-F., et al. (2018). Antitumor activity of EGFR-specific CAR T cells against non-small-cell lung cancer cells in vitro and in mice. Cell Death Dis. *9*, 177.

11. Guo, X., Jiang, H., Shi, B., Zhou, M., Zhang, H., Shi, Z., Du, G., Luo, H., Wu, X., Wang, Y., et al. (2018). Disruption of PD-1 Enhanced the Anti-tumor Activity of Chimeric Antigen Receptor T Cells Against Hepatocellular Carcinoma. Front. Pharmacol. *9*, 1118.

12. Hung, C.F., Xu, X., Li, L., Ma, Y., Jin, Q., Viley, A., Allen, C., Natarajan, P., Shivakumar, R., Peshwa, M.V., and Emens, L.A. (2018). Development of Anti-Human Mesothelin-Targeted Chimeric Antigen Receptor Messenger RNA-Transfected Peripheral Blood Lymphocytes for Ovarian Cancer Therapy. Hum. Gene Ther. *29*, 614–625.

13. Junghans, R.P., Ma, Q., Rathore, R., Gomes, E.M., Bais, A.J., Lo, A.S.Y., Abedi, M., Davies, R.A., Cabral, H.J., Al-Homsi, A.S., and Cohen, S.I. (2016). Phase I Trial of Anti-PSMA Designer CAR-T Cells in Prostate Cancer: Possible Role for Interacting Interleukin 2-T Cell Pharmacodynamics as a Determinant of Clinical Response. Prostate *76*, 1257–1270.

14. Hou, B., Tang, Y., Li, W., Zeng, Q., and Chang, D. (2019). Efficiency of CAR-T Therapy for Treatment of Solid Tumor in Clinical Trials: A Meta-Analysis. Dis. Markers *2019*, 3425291.

15. Martinez, M., and Moon, E.K. (2019). CAR T Cells for Solid Tumors: New Strategies for Finding, Infiltrating, and Surviving in the Tumor Microenvironment. Front. Immunol. *10*, 128.

16. Klebanoff, C.A., Rosenberg, S.A., and Restifo, N.P. (2016). Prospects for gene-engineered T cell immunotherapy for solid cancers. Nat. Med. *22*, 26–36.

17. D'Aloia, M.M., Zizzari, I.G., Sacchetti, B., Pierelli, L., and Alimandi, M. (2018). CAR-T cells: the long and winding road to solid tumors. Cell Death Dis. *9*, 282.

18. Bernhard, H., Neudorfer, J., Gebhard, K., Conrad, H., Hermann, C., Nährig, J., Fend, F., Weber, W., Busch, D.H., and Peschel, C. (2008). Adoptive transfer of autologous, HER2-specific, cytotoxic T lymphocytes for the treatment of HER2-overexpressing breast cancer. Cancer Immunol. Immunother. *57*, 271–280.

19. Mulligan, A.M., Raitman, I., Feeley, L., Pinnaduwage, D., Nguyen, L.T., O'Malley, F.P., Ohashi, P.S., and Andrulis, I.L. (2013). Tumoral lymphocytic infiltration and expression of the chemokine CXCL10 in breast cancers from the Ontario Familial Breast Cancer Registry. Clin. Cancer Res. *19*, 336–346.

20. Harlin, H., Meng, Y., Peterson, A.C., Zha, Y., Tretiakova, M., Slingluff, C., McKee, M., and Gajewski, T.F. (2009). Chemokine expression in melanoma metastases associated with CD8+ T-cell recruitment. Cancer Res. *69*, 3077–3085.

21. Förster, R., Emrich, T., Kremmer, E., and Lipp, M. (1994). Expression of the G-protein–coupled receptor BLR1 defines mature, recirculating B cells and a subset of T-helper memory cells. Blood *84*, 830–840.

22. Fazilleau, N., Mark, L., McHeyzer-Williams, L.J., and McHeyzer-Williams, M.G. (2009). Follicular helper T cells: lineage and location. Immunity *30*, 324–335.

23. Förster, R., Mattis, A.E., Kremmer, E., Wolf, E., Brem, G., and Lipp, M. (1996). A putative chemokine receptor, BLR1, directs B cell migration to defined lymphoid organs and specific anatomic compartments of the spleen. Cell *87*, 1037–1047.

24. Wang, G.-Z., Cheng, X., Zhou, B., Wen, Z.-S., Huang, Y.-C., Chen, H.-B., Li, G.-F., Huang, Z.-L., Zhou, Y.-C., Feng, L., et al. (2015). The chemokine CXCL13 in lung cancers associated with environmental polycyclic aromatic hydrocarbons pollution. Elife *4*, e09419.

25. Singh, R., Gupta, P., Kloecker, G.H., Singh, S., and Lillard, J.W., Jr. (2014). Expression and clinical significance of CXCR5/CXCL13 in human non-small cell lung carcinoma. Int. J. Oncol. *45*, 2232–2240.

26. Feng, K., Guo, Y., Dai, H., Wang, Y., Li, X., Jia, H., and Han, W. (2016). Chimeric antigen receptor-modified T cells for the immunotherapy of patients with EGFR-expressing advanced relapsed/refractory non-small cell lung cancer. Sci. China Life Sci. *59*, 468–479.

27. Guo, Y., Feng, K., Liu, Y., Wu, Z., Dai, H., Yang, Q., Wang, Y., Jia, H., and Han, W. (2018). Phase I Study of Chimeric Antigen Receptor-Modified T Cells in Patients with EGFR-Positive Advanced Biliary Tract Cancers. Clin. Cancer Res. *24*, 1277–1286.

28. Di Stasi, A., De Angelis, B., Rooney, C.M., Zhang, L., Mahendravada, A., Foster, A.E., Heslop, H.E., Brenner, M.K., Dotti, G., and Savoldo, B. (2009). T lymphocytes coexpressing CCR4 and a chimeric antigen receptor targeting CD30 have improved homing and antitumor activity in a Hodgkin tumor model. Blood *113*, 6392–6402.

29. Craddock, J.A., Lu, A., Bear, A., Pule, M., Brenner, M.K., Rooney, C.M., and Foster, A.E. (2010). Enhanced tumor trafficking of GD2 chimeric antigen receptor T cells by expression of the chemokine receptor CCR2b. J. Immunother. *33*, 780–788.

30. Moon, E.K., Carpenito, C., Sun, J., Wang, L.-C.S., Kapoor, V., Predina, J., Powell, D.J., Jr., Riley, J.L., June, C.H., and Albelda, S.M. (2011). Expression of a functional CCR2 receptor enhances tumor localization and tumor eradication by retargeted human T cells expressing a mesothelin-specific chimeric antibody receptor. Clin. Cancer Res. *17*, 4719–4730.

31. Haran, K.P., Hajduczki, A., Pampusch, M.S., Mwakalundwa, G., Vargas-Inchaustegui, D.A., Rakasz, E.G., Connick, E., Berger, E.A., and Skinner, P.J. (2018). Simian Immunodeficiency Virus (SIV)-Specific Chimeric Antigen Receptor-T Cells Engineered to Target B Cell Follicles and Suppress SIV Replication. Front. Immunol. *9*, 492.

32. Jin, L., Cao, L., Zhu, Y., Cao, J., Li, X., Zhou, J., Liu, B., and Zhao, T. (2021). Enhance anti-lung tumor efficacy of chimeric antigen receptor-T cells by ectopic expression of C–C motif chemokine receptor 6. Sci. Bull. *66*, 803–812.

33. Zheng, Z., Cai, Y., Chen, H., Chen, Z., Zhu, D., Zhong, Q., and Xie, W. (2019). CXCL13/CXCR5 Axis Predicts Poor Prognosis and Promotes Progression Through PI3K/AKT/mTOR Pathway in Clear Cell Renal Cell Carcinoma. Front. Oncol. *8*, 682.

OMTO, Volume *22*

Supplemental information

CXCR5 guides migration and tumor eradication of anti-EGFR chimeric antigen receptor T cells

Guangchao Li, Jintao Guo, Yanfang Zheng, Wen Ding, Zheping Han, Lingyu Qin, Wenjun Mo, and Min Luo

Supplementary Figure 1

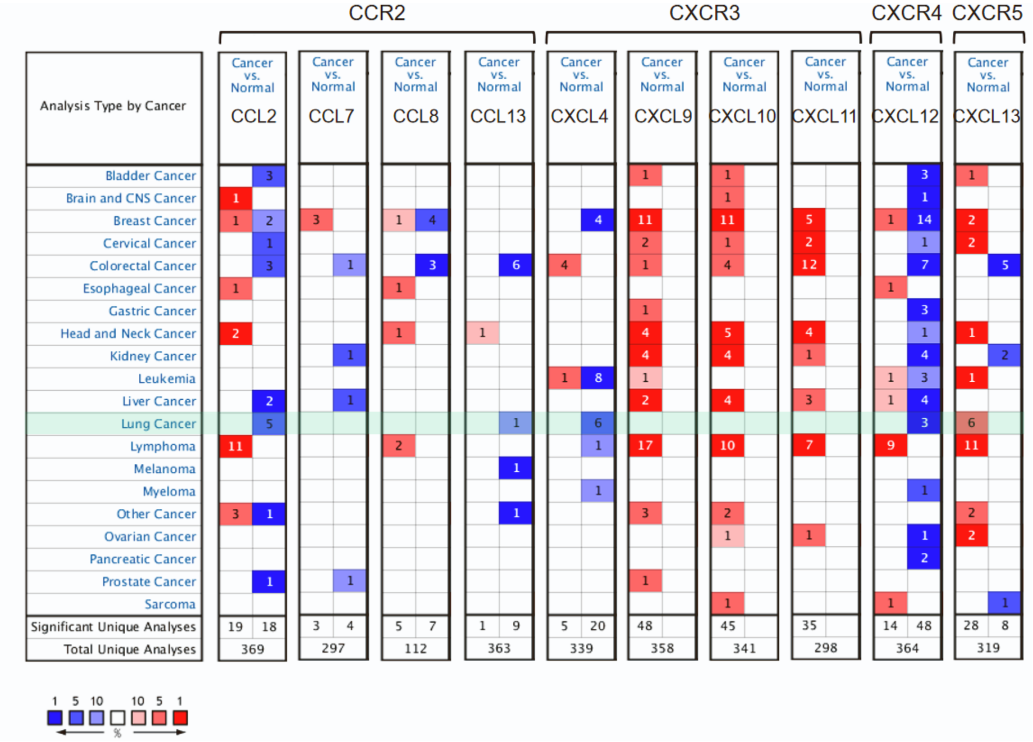

Figure S1. The expressions of multiple chemokines in the normal and lung cancer.

The mRNA expression levels of chemokines in various cancers were analyzed using the online Oncomine database (Threshold for FC>2, $p<0.05$, and rank=top10).

Supplementary Figure 2

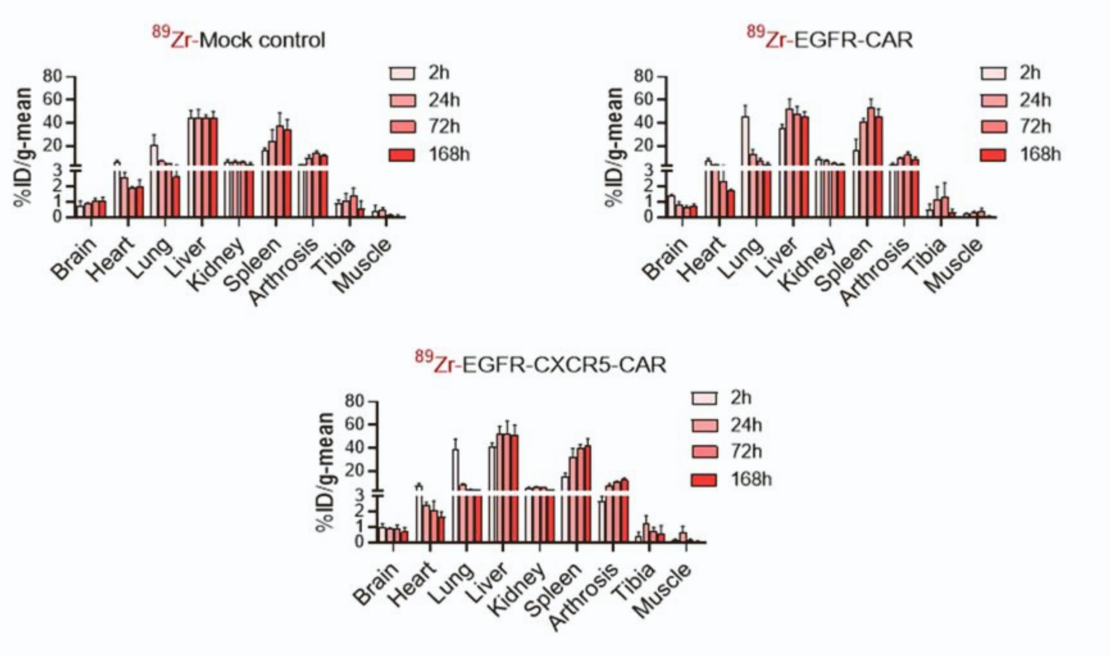

Figure S2. Micro PET/CT analysis of the 89Zr-Oxine labeled CAR-T cells tracking in vivo.

After a single intravenous injection of ^{89}Zr-CAR-T cells in tumor-bearing mice, a small animal PET/CT scan was performed to delineate the regions of interest and obtain the radiation uptake values to study the tissue distribution characteristics of CAR-T cells. Radio-isotopes were imaged on tumor bearing mice over 7 days (n=3).
